# Risk factors and outcomes of postoperative pancreatic fistula after pancreatico-duodenectomy: an audit of 532 consecutive cases

**DOI:** 10.1186/s12893-015-0011-7

**Published:** 2015-03-26

**Authors:** Shun-Jun Fu, Shun-Li Shen, Shao-Qiang Li, Wen-Jie Hu, Yun-Peng Hua, Ming Kuang, Li-Jian Liang, Bao-Gang Peng

**Affiliations:** Department of Hepatobiliary Surgery, the First Affiliated Hospital, Sun Yat-sen University, Guangzhou, 510080 PR China; Department of Hepatobiliary Surgery, The Second Affiliated Hospital of Guangzhou University of Chinese Medicine (Guangdong Provincial Hospital of TCM), Guangzhou, 510120 PR China

**Keywords:** Pancreatic fistula, Pancreaticoduodenectomy, Risk factor, Outcome

## Abstract

**Background:**

Pancreatic fistula (PF) remains the most challenging complication after pancreaticoduodenectomy (PD). The purpose of this study was to identify the risk factors of PF and delineate its impact on patient outcomes.

**Methods:**

We retrospectively reviewed clinical data of 532 patients who underwent PD and divided them into PF group and no PF group. Risk factors and outcomes of PF following PD were examined.

**Results:**

PF was found in 65 (12.2%) cases, of whom 11 were classified into ISGPF grade A, 42 grade B, and 12 grade C. Clinically serious postoperative complications in the PF versus no PF group were mortality, abdominal bleeding, bile leak, intra-abdominal abscess and pneumonia. Univariate and multivariate analysis showed that blood loss ≥ 500 ml, pancreatic duct diameter ≤ 3 mm and pancreaticojejunostomy type were independent risk factors of PF after PD.

**Conclusions:**

Blood loss ≥ 500 ml, pancreatic duct diameter ≤ 3 mm and pancreatico-jejunostomy type were independent risk factors of PF after PD. PF was related with higher mortality rate, longer hospital stay, and other complications.

## Background

Pancreaticoduodenectomy (PD) is the classic surgical approach for both malignant and benign disease of pancreatic head, duodenum and periampullary region. PD was associated with high mortality and morbidity since introduced as a treatment approach. Recently, both the mortality and morbidity following PD were significantly reduced for the improvement of surgical skills and perioperative care. The mortality rate after PD was kept even as low as 5% in many specialized centers [1-4]. Although the mortality following PD has decreased, the morbidity rate remains at a high level [1,4,5], with pancreatic fistula (PF) as the most challenging complication. Risk factors for PF after PD described in the literature included patient demographics, diameter of pancreatic duct, pancreatic texture and anastomotic technique [5-8]. Generally, PF is correlated with other major complications such as intraperitoneal bleeding, abdominal abscess, delayed gastric emptying and wound infection, all of which prolong the hospital stays and increase health-care costs [6-8].

To identify patients at high risk for PF and avoid the accompanying complications, it is important to predict its occurrence so that prompt treatment can be administered. The purpose of this study was to analyze the risk factors and outcomes of PF from a prospective database of 532 patients undergoing PD.

## Methods

Five hundred thirty-two patients undergoing elective PD in the first Affiliated Hospital of Sun Yat-sen University between April 2000 and November 2011 were included in this study. Clinical data including demographics, intraoperative procedures and postoperative complications were reviewed. Prior approval from the committee for Ethical Review of research involving Human Subject of the first Affiliated Hospital of Sun Yat-sen University (Guangzhou, China) and informed consent from patients were obtained before study. One hundred and forty-one patients (26.5%, 141/532) accepted preoperative biliary drainage.

All patients were divided into 2 groups, according to the presence of PF or not: PF group and no PF group. PF, delayed gastric emptying (DGE), and post-pancreatectomy hemorrhage (PPH) were defined according to the International Study Group on Pancreatic Fistula (ISGF) [9-11]. Bile leak was diagnosed based on the following criteria: (1) bile fluid in the peritoneal drainage or oozing from the wound; or (2) radiographically proven fluid collection requiring percutaneous drainage and demonstrating elevated bilirubin levels [12,13]. The operative mortality was defined as death within 30 days after operation or before discharge from the hospital (irrespective of the duration of stay). Other morbidities were defined as any of the following: intraabdominal abscess (IAA) (drainage from an intraoperatively placed drain with positive cultures requiring the placement of a new percutaneous drain or operative intervention); pneumonia (fever, leukocytosis, culture-positive sputum with polymorphonuclear leukocytes on Gram stain, and chest radiograph demonstrating focal infiltrates); wound infection (purulent drainage from the postoperative wound, requiring opening and packing of the wound), deep venous thrombosis (characteristic venous obstruction, demonstrated on Doppler ultrasound), chyle leak (milky drainage from the peritoneal drain) [13]. Postoperative complications were graded according to Clavien-Dindo classification [5].

### Surgical technique

Of the 532 patients, 7 cases had a pylorus-preserving pancreaticoduodenectomy (PPPD) and the others had a classical pancreaticoduodenectomy (PD). The pancreaticojejunostomy was performed using a traditional 2-layer anastomosis (n = 304), an invaginated anastomosis (n = 125), and a duct-mucous anastamosis (n = 103) based on surgeon’s preference. No pancreaticogastrostomy was used in our study. Four hundred and seventeen anastomoses (78.4%) were constructed using pancreatic duct stents. The biliary stent was not routinely placed. Drains were placed routinely in the Winslow’s foramen for draining peritoneal fluid in all patients. Prophylactic octreotide or somatostatin was not routinely used.

### Statistical analysis

All statistical analyses were performed with SPSS version 21.0 software (IBM, USA). Quantitative data are expressed as mean ± SD. Independent sample Student’s *t* test was used to compare continuous variables. The Chi-square test or Fisher exact test was used to compare categorical variables. Of all the variables tested in univariate analysis, only those with *P* < .05 were put into multivariate analysis. Logistic regression (forward selection) was performed to determine the independent risk factors of PF. *P* < .05 was considered statistically significant.

## Results

### Incidence of pancreatic fistula

The indications for PD included duodenal neoplasm, pancreatic cancer, bile duct neoplasm, ampullary neoplasm, chronic pancreatitis, gastric antrum cancer, cystadenoma, pancreatic cyst, and so on. Three major indications for PD were duodenal neoplasm in 164 patients (30.8%), pancreatic cancer in 140 patients (26.3%) and ampullary neoplasm in 102 patients (19.2%). Sixty-five patients (12.2%) developed PF after PD, A grade 11 cases (16.9%, 11/65), B grade 42 cases (64.6%, 42/65) and C grade 12 cases (18.5%, 12/65).

### Risk factors of pancreatic fistula

Fourteen clinical factors of PF were compared between the PF group and the no PF group by univariate analysis. We found that blood loss ≥ 500 ml, pancreatic duct diameter ≤ 3 mm and pancreaticojejunostomy type had statistical significance. Multivariate logistic regression analysis was used to identify the independent risk factors of PF. We identified that blood loss ≥ 500 ml (hazard ratio [HR] = 2.281; 95% confidence interval [CI] 1.334-3.901; *P* = .003), pancreatic duct diameter ≤ 3 mm (HR = 0.351; 95%CI, 0.192-0.641; *P* = .001) and pancreaticojejunostomy type (HR = 1.355; 95%CI, 1.007-1.823; *P* = .045) were independent risk factors for PF after PD (Table 1).

**Table 1:** **Risk factors of pancreatic fistula by univariate and multivariate analysis**

**Variable**	**PF group**	**NPF group**	**t/X** ^**2**^	***P***	**HR**	**95% CI**	***P***
Total	65	467					
Preoperative factors							
Age (years)	58.09 ± 11.50	55.48 ± 12.10	1.640	0.102			
Gender							
Male	46(70.8%)	298(63.8%)					
Female	19(29.2%)	169(36.2%)	1.209	0.272			
Hemoglobin serum levels (g/L)	117.85 ± 24.05	120.03 ± 20.86	0.696	0.487			
Albumin			0.075	0.784			
<35 g/L	9(13.8%)	59(12.6% )					
≥35 g/L	56(86.2)	408(87.4%)					
Hypertension			1.545	0.214			
Yes	11(16.9%)	54(11.6%)					
No	54(83.1%)	413(88.4%)					
Diabetes			0.015	0.903			
Yes	7(10.8%)	48(10.3%)					
No	58(89.2%)	419(89.7%)					
Preoperative biliary drainage			0.005	0.943			
With	17(26.2%)	124(26.6%)					
Without	48(73.8%)	343(73.4%)					
Intraopertive factors							
Operative time(min)	378.15 + 91.36	356.97 + 84.87	1.868	0.062			
Blood loss			0.075	0.784			
<500 ml	28(43.1%)	293(62.7%)	9.219	0.002	2.281	1.334-3.901	0.003
≥500 ml	37(56.9%)	174(37.3%)					
Pancreatic texture			2.064	0.151			
Soft	54(83.1%)	350(74.9%)					
Firm	11(16.9%)	117(25.1%)					
Diameter of pancreatic duct			14.643	<0.001	0.351	0.192-0.641	0.001
≤3 mm	49(75.4%)	234(50.1%)					
>3 mm	16(24.6%)	233(49.9%)					
Pancreatic stent			0.435	0.510			
Yes	53(81.5%)	364(77.9%)					
No	12(18.2%)	103(22.1%)					
Preoperative biliary drainage			0.005	0.943			
With	17(26.2%)	124(26.6%)					
Without	48(73.8%)	343(73.4%)					
Intraopertive factors							
Operative time(min)	378.15 + 91.36	356.97 + 84.87	1.868	0.062			
Blood loss			0.075	0.784			
<500 ml	28(43.1%)	293(62.7%)	9.219	0.002	2.281	1.334-3.901	0.003
≥500 ml	37(56.9%)	174(37.3%)					
Pancreatic texture			2.064	0.151			
Soft	54(83.1%)	350(74.9%)					
Firm	11(16.9%)	117(25.1%)					
Diameter of pancreatic duct			14.643	<0.001	0.351	0.192-0.641	0.001
≤3 mm	49(75.4%)	234(50.1%)					
>3 mm	16(24.6%)	233(49.9%)					
Pancreatic stent			0.435	0.510			
Yes	53(81.5%)	364(77.9%)					
No	12(18.2%)	103(22.1%)					
Venous resection			1.095	0.295			
Yes	3(4.6%)	39(8.4%)					
No	62(95.4%)	428(91.6%)					
PJ types			15.992	<0.001	1.355	1.007-1.823	0.045
Traditional 2-layers anastomosis	33(50.8%)	271(58.0%)					
Invaginated anastomosis	27(41.5%)	98(21.0%)					
Duct to mucous anastomosis	5(7.7%)	98(21.0%)					

*PF*: pancreatic fistula; *NPF*: non pancreatic fistula; *PJ*: pancreaticojejunostomy; *HR*: hazard ratio; *CI*: confidence interval.

### Postoperative outcomes

A total of 303 (57.0%, 303/532) patients didn’t develop any complication, while 229 (43.0%, 229/532) patients had at least one complication: 60 (11.3%, 60/532) patients had grade Icomplications, 50 (9.4%, 50/532) patients had grade II complications, 93 (17.5%, 93/532) patients had grade III complications, 3 (0.6%, 3/532)patients had grade IV complications. Grade V (death) occurred in 23 patients (4.3%, 23/532). Besides PF, other postoperative complications included PPH (60/532, 11.3%), bile leak (17/532, 3.2%), delayed gastric emptying (18/532, 3.4%), intraabdominal abscess (IAA) (97/532, 18.2%), pneumonia (27/532, 5.1%), wound infection (88/532, 16.5%), deep venous thrombosis (DVT) (1/532, 0.2%) and chyle leak (2/532, 0.4%). Mortality, IAB, bile leak, IAA and pneumonia were significantly higher in the PF group when compared with the no PF group. The length of hospital stay significantly increased in those patients who developed PF compared with those that did not (Table 2).

**Table 2:** **Postoperative outcomes between the two groups**

**Outcomes**	**PF group (n = 65)**	**NPF group n = 467**	**t/X** ^**2**^	***P***
Mortality	7/58(10.8%)	16/451(3.4%)	7.438	0.006
LOS	50.62 ± 30.16	30.19 ± 15.44	8.633	<0.001
Abdominal bleeding	6/59(9.2%)	10/457(2.1%)	9.831	0.002
Gastrointestinal bleeding	9/56(13.8%)	38/429(8.1%)	2.309	0.129
Bile leak	5/60(7.7%)	12/455(2.6%)	4.840	0.028
DGE	4/61(6.2%)	14/453(3.0%)	1.465	0.187
IAA	50/15(76.5%)	47/420(10.1%)	171.08	<0.001
pneumonia	7/58(10.8%)	20/447(4.3%)	4.983	0.026
Wound infection	9/56(13.8%)	79/388(16.9%)	0.390	0.532
DVT	0(0)	1/466(0.21%)	0.139	0.709
chyle leak	0(0)	2/465(0.43%)	0.279	0.597
Repeat operation	9(13.85%)	15/452(3.21%)	14.979	<0.001

*LOS*: length of stay; *DGE*: delayed gastric emptying; *IAA*: intraabdominal abscess; *DVT*: deep venous thrombosis; Other abbreviations as in Table 1.

## Discussion

PF is the most challenging complication after PD. Because it was associated with substantial mortality and morbidity, some authors even defined it as an “Achilles heel” of PD [6]. Our results demonstrate that the incidence of PF after PD was about 12.0%, which appears to be comparable to the PF rate of 9–14% reported in other specialized centers [14-17].

The formation of PF is associated with many variables involving demographic, preoperative, intraoperative and pathological factors. Previous studies had reported that pancreaticogastrostomy was associated with significantly lower pancreatic fistula rate than pancreaticojejunostomy. Pancreaticogastrostomy was more efficient than pancreatico-jejunostomy in reducing the incidence of postoperative pancreatic fistula [18-23]. In our institution, we have not performed pancreaticogastrostomy after PD so far. There is still a controversy in the relationship between preoperative biliary drainage and the incidence of PF. A study from Hopkins suggested that preoperative stent was associated with high incidence of postoperative pancreatic fistula [24]. Schmidt *et al.* also showed that those patients who had preoperative percutaneous transhepatic biliary (PTB) stent showed an increased risk of PF formation [6]. However, Aranha *et al.* found that no difference in the incidence of PF versus no-PF in those patients having ERCP or PTC and an increase in PF in those patients who did not have preoperative stents compared with those patients who did [8]. Lin *et al.* indicated that no difference in the incidence of PF versus no-PF in those patients having preoperative stent (either endoscopic or PBD) [14]. Our results also showed that there was no relationship between preoperative biliary drainage and postoperative PF.

As expected, increased intraoperative blood loss appeared to be an important risk factor of PF. Yeh *et al.* demonstrated increased intraoperative blood loss was an independent risk factor for PF after PD by univariate and multivariate analysis [25]. The factors which might increase blood loss during operation included a more advanced stage of the disease such as portal vein invasion or superior mesenteric vein, adhesions due to prior operations, jaundice-associated coagulopathy, obesity, and concurrent pancreatitis [25]. In our study, Intraoperative blood loss exceeding 500 ml occurred in 39.7% (211 of 532 patients) of patients, and of those, 17.5% patients (37/211) developed PF, which was significantly higher than patients with blood loss less than 500 ml (28/321, 8.7%). Again, our results showed blood loss ≥ 500 ml (hazard ratio [HR] = 2.281; 95% confidence interval [CI] 1.334-3.901; *P* = .003) was an independent risk factor for PF after PD. Kawai *et al.* showed that the preoperative CT image-assessed ligation of inferior pancreaticoduodenal artery (IPDA) method (CLIP) was a useful and reliable operative technique for reducing intraoperative bleeding in PD [26]. Ishizaki *et al.* reported early ligation of the inferior pancreatoduodenal artery (IPDA) not only reduced intraoperative blood loss during PD but also alleviated postoperative morbidity and mortality [27]. Therefore, IPDA method was suggested to decrease intraoperative bleeding.

Type of pancreaticojejunostomy anastomosis has also been cited as a predictor of PF after PD. Berger *et al.* reported considerably fewer fistulas with invagination compared with duct to mucosapancreaticojejunostomy after pancreaticoduodenectomy by a randomized, prospective, dual-institution trial [28]. However, Schmidt *et al.* and Bartoli *et al.* reported that lowest incidence of PF in patients who had a duct to mucous anastomosis than other anastomoses [6,29]. We also found that the lowest incidence of PF in patients who had a duct to mucous anastomosis (4.9%) versus a traditional 2-layers pancreaticojejunostomy (10.9%) or an invaginated pancreaticojejunostomy (21.6%) (*P* < .001). Since the diameter of major pancreatic duct played an important role in selection of PJ types, some studies have concluded that major pancreatic duct diameter was also an independent risk factor of PF after PD [4,23]. Generally, the narrowed pancreatic duct diameter is not only more challenging to reconstruct, but also more likely to either occlude or dehisce. In our study, we found that PF in patients who had pancreatic duct diameter > 3 mm (6.4%) was significantly lower than those who had pancreatic duct diameter ≤ 3 mm (17.3%) (*P* < .001). Soft pancreatic tissue has been regarded as a potent contributor for PF formation [4,7,14], while it had no effect on PF in our study. Based on our results and literatures, we think we should try to adopt duct to mucous anastomosis when the pancreatic duct diameter > 3 mm. If the pancreatic duct diameter measured 3 mm or less, we can expand the pancreatic duct by placing a fine stay suture in the middle of the anterior wall of the pancreatic duct as described by Hatori [30] and performed pancreaticojejunostomy with duct-to-mucosa anastomosis without a stent tube [31].

PF is associated with a greater many of other complications and prolonged hospital stay [6,8,14,28]. In our study, it was found to be associated with mortality, abdominal bleeding, bile leakage, intraabdominal abscess, pneumonia, reoperations and long hospital stay. Other complications including gastrointestinal bleeding, delayed gastric emptying, wound infection, deep venous thrombosis (DVT) and chyle leak were not significant when the PF group was compared with the no PF group.

## Conclusions

In conclusion, blood loss and the pancreatic duct diameter and pancreaticojejunostomy type were independent risk factors for PF after PD. Outcomes in patients with PF were remarkable for a higher rate of mortality, abdominal bleeding and septic complications, an increased incidence of reoperations, and a longer hospital stay.

## Abbreviations

PF: Pancreatic fistula
PD: Pancreaticoduodenectomy
DEG: Delayed gastric emptying
PPH: Post-pancreatectomy hemorrhage
ISGF: International study group on pancreatic fistula
IAA: Intraabdominal abscess
PPPD: Pylorus-preserving pancreaticoduodenectomy
HR: Hazard ratio
CI: Confidence interval
DVT: Deep venous thrombosis
PTB: Percutaneous transhepatic biliary
IPDA: Inferior pancreaticoduodenal artery
